# Sialylated human milk oligosaccharides prevent intestinal inflammation by inhibiting toll like receptor 4/NLRP3 inflammasome pathway in necrotizing enterocolitis rats

**DOI:** 10.1186/s12986-020-00534-z

**Published:** 2021-01-06

**Authors:** Wenting Zhang, Jingqiu He-Yang, Wenjuan Tu, Xiaoying Zhou

**Affiliations:** 1 Department of Pharmacy, Affiliated Changzhou Children’s Hospital of Nantong University, Changzhou, 213003 Jiangsu China; 2 Immunopharmacology Institute, School of Pharmacy, School of Medicine, Changzhou University, Changzhou, 213164 Jiangsu China; 3 Department of Neonatology, Affiliated Changzhou Children’s Hospital of Nantong University, Changzhou, 213003 Jiangsu China

**Keywords:** Necrotizing enterocolitis, Human milk oligosaccharide, Neonates, Nod-like receptor pyrin domain-containing 3, Inflammation

## Abstract

**Background:**

Necrotizing enterocolitis (NEC) remains a fatal gastrointestinal disorder in neonates and has very limited therapeutic options. Sialylated human milk oligosaccharides (SHMOs) improve pathological changes in experimental NEC models. The objectives of this study were to investigate the involvement of NLRP3 inflammasome in NEC pathology and to explore the effects of SHMOs on toll-like receptor 4 (TLR4)/nuclear factor κB (NF-κB)/NLRP3 inflammatory pathway in experimental NEC.

**Methods:**

The intestinal-tissue segments were collected from NEC infants, NLRP3 and caspase-1 positive cell were examined by immunohistochemistry. Newborn rats were hand-fed with formula containing or non-containing SHMOs (1500 mg/L) and exposed to hypoxia/cold stress to induce experimental NEC. The NEC pathological scores were evaluated; ileum protein expression of membrane TLR4 (mTLR4), inhibitor κB-α (IκB-α), NF-κB p65 subunit and phospho-NF-κB p65, as well as NLRP3 and caspase-1 were analyzed; ileum concentrations of interleukin-1β, interleukin-6, tumor necrosis factor-α (TNF-α) were also measured. Human colon epithelial Caco-2 cells were pre-treated with or without SHMOs and stimulated with TLR4 activator, lipopolysaccharide. Cell viabilities, mitochondrial membrane potential and supernatant matrix metalloprotease 2 (MMP-2) activities were analyzed.

**Results:**

Increased frequencies of NLRP3 and caspase-1 positive cells were found in the lamina propria of damaged intestinal area of NEC neonates. SHMOs supplementation reduced NEC incidence and pathological damage scores of rats challenged with hypoxia/cold stress. Accumulation of interleukin-1β, interleukin-6 and TNF-α in NEC group were attenuated in SHMOs + NEC group. Protein expression of mTLR4, NLRP3 and caspase-1 were elevated, cytoplasmic IκB-α were reduced, nuclear phospho-NF-κB p65 were increased in the ileum of NEC rats. SHMOs supplementation ameliorated the elevation of mTLR4, NLRP3 and caspase-1, restored IκB-α in the cytoplasmic fraction and reduced phospho-NF-κB p65 in the nuclear fraction in the ileum of NEC rats. SHMOs pre-treatment improved Caco-2 cell viability, mitigated loss of mitochondrial membrane potential and modulated MMP-2 activities in the presence of lipopolysaccharide in-vitro.

**Conclusions:**

This study provided clinical evidence of involvement of NLRP3 inflammasome in NEC pathology, and demonstrated the protective actions of SHMOs might be owing to the suppression of TLR4/NF-κB/NLRP3-mediated inflammation in NEC.

## Background

Necrotizing enterocolitis (NEC) is one of the most common and devastating diseases in neonates [1]. It occurs in approximately 0.34% of live births and affects up to 10% of premature infants born below 32 weeks' gestation [2, 3]. The estimated rate of death related to NEC ranges between 20 and 30%, with the highest rate in infants requiring surgical intervention [4, 5]. Despite decades of intensive research, the precise etiology remains unclear, fully preventive and therapeutic approaches are still limited [6].

Human milk feeding is long known to reduce the incident of NEC in preterm infants, while formula milk does the opposite [7]. Human milk contains many bioactive compounds including a diverse repertoire of human milk oligosaccharides (HMOs). The current opinion is that HMOs are not digested in the proximal intestine, they reach the small intestine and colon intact, and function as prebiotics for intestinal bacterial flora or intestinal immune regulator, and thus enhancing intestinal barrier homeostasis and protecting from pathogen invasion [8]. In human milk, about 50–80% of HMOs are fucosylated, 20–30% are sialylated [8]. It has been reported that sialylated human milk oligosaccharides (SHMOs) improved NEC symptoms in a rodent model of NEC, and this protection was abolished following neuraminidase-treatment [9]. Further investigations showed that synthetic disialyl hexasaccharides and enzymatically sialylated galacto-oligosaccharides (GOS), but not unmodified GOS, were capable of reducing the pathological scores in NEC rats [10, 11]. These evidences suggested sialylation is a required modification of oligosaccharides exerting their anti-NEC actions. Current studies exploring beneficial effects of SHMOs have shown that SHMOs interfere with host-microbial interactions by acting as antiadhesive antimicrobials or specific probiotic growth promoting agents, and also directly regulate host intestinal epithelial responses by modulating gene expression of sialyltransferases on epithelial cell surface glycans, thereby preventing the binding of enteropathogenic *Escherichia coli* to mucosal epithelium [12–14]. However, the protective mechanisms of SHMOs in NEC remain unclear.

NEC develops as a consequence of hyper-active inflammatory processes triggered by several perinatal insults including formula milk feeding, bacterial colonization, toll like receptor 4 (TLR4) hyper-expression and hypoxic stress [15]. TLR4 is of specific interest in NEC pathogenesis. In gastrointestinal tract, TLR4 is widely expressed in epithelial cells, endothelial cells, fibroblasts and several immune granulocytes such as macrophages, mast cells and dendritic cells [16, 17]. High expression of TLR4 has been found in fetal colonic epithelium of human and mouse and in intestinal samples from NEC infants [18, 19]. Hyper-expression of TLR4 in different cell types triggers different biological consequences: epithelial TLR4 hyper-responsiveness is responsible for disruption of epithelium integrity and initiation and perpetuation of inflammatory responses [20]; endothelial TLR4 overexpression impairs intestinal microcirculatory perfusion via endothelial nitric oxide synthase signalling [21]; TLR4 activation in fetal intestinal fibroblasts causes a significant inflammatory response [16]. These events work together and proceed NEC development. NEC initiates with disrupted integrity of intestinal epithelial layer. Mechanically, TLR4 activation promotes apoptosis and inhibits proliferation of enterocytes, and also alters enterocytes migration throughout crypt-villus axis by modulating interaction between cell and extracellular matrix [15, 22]. Another pathological mechanism of TLR4 includes a hyper-responsiveness to microbial ligands upon pathological and commercial bacteria mediated by TLR4 overexpression in premature gut [20]. A critical link between TLR4 hyper-expression and overt inflammatory disorder could be downstream interleukin-1β (IL-1β) production. IL-1β can induce intestinal inflammation and play a regulatory role in epithelial healing and repairing processes via recruitment and activation of immune cells and through triggering production of chemokines, pro-inflammatory cytokines, and growth factors [23]. Maturation and secretion of IL-1β is highly controlled by TLR4/nuclear factor-κB (NF-κB)-induced activation of nod-like receptor pyrin domain-containing 3 (NLRP3) inflammasome [24, 25], in order to prevent inappropriate inflammation. NLRP3 inflammasome activation requires two-step process: NLRP3 priming process following stimulations such as bacterial lipopolysaccharide (LPS) and subsequent NLRP3 activating process. On activation, NLRP3 assembles into a multi-protein inflammasome complex, causing caspase-1 autoproteolysis and subsequent pro-cytokines (mainly IL-1β and IL-18) cleavage to their mature forms. A previous study found that the protein and mRNA expression of NLRP3 were increased in NEC rats [26]. However, related information especially from clinical evidence regarding the involvement of TLR4/NLRP3 pathway in NEC pathology is lacking; the role of human milk in regulating this pathway has not been well explored.

Along with lines mentioned above, this study was conducted to probe involvement of NLRP3 in the pathology of NEC and investigate the protective mechanism of SHMOs along TLR4/NF-κB/NLRP3 signaling pathway. For this purpose, inflamed intestinal biopsies from neonatal NEC infants was examined; a neonatal rat model of NEC and LPS-stimulated human colonic epithelial cell model were established; SHMOs were separated from human breast milk and applied to the animal and cell models.

## Methods and procedures

### Ethical declaration

This study involving human biopsies and animal experiment protocols was approved by the Ethics Committee of Affiliated Changzhou Children’s Hospital of Nantong University and Institutional Animal Care and Use Committee of Nantong University (20180022). All the human biopsies were obtained with the approval and supervision of Department of Pathology of Affiliated Changzhou Children’s Hospital of Nantong University. All efforts were made to minimize animal suffering.

### Histomorphological and immunohistochemical staining

The ileum segments embedded paraffin blocks were obtained from 13 neonatal infants (postnatal age range, 1–28 days) who needed surgeries, including 8 with NEC and 5 with intestinal atresia. Histomorphological examination was performed using haematoxylin and eosin (HE) staining; immunohistochemical (IHC) analysis were performed by BenchMark GX automated stainer (Roche). Rabbit polyclonal anti-NLRP3 (1:200, Abcam, UK) and mouse monoclonal anti-caspase-1 antibodies (1:50, Santa Cruz, USA) were used as primary antibodies. The horseradish peroxidase (HRP)-labelled secondary antibodies were used accordingly. Ventana UltraView Universal diaminobenzidine (DAB) detection kit (Ventana Medical Systems, Inc.) was used for visualization, and photomicrographs were obtained with an inverted microscope (Olympus, USA) at 100× or 400×.

### SHMOs separation from human breast milk

Pooled SHMOs were isolated and fractionated from about 20 L of human milk collected from 20 healthy mothers after the written informed consent was obtained. The lipid layer was removed by 10-min centrifugation at 8000 rpm at 4 °C, and the proteins were precipitated from the aqueous phase by adding ethanol (1:2, v/v). HMOs-containing supernatant was concentrated and freeze-dried. Pooled HMOs were fractionated using a preparative hydrophilic interaction liquid chromatography (prep-HILIC, 250 mm × 50 mm). Lyophilized HMOs-containing power was dissolved in methanol/water (1:1, v/v) and injected into a pre-HILIC system. The mobile phase was ethanol/water with a gradient condition: 0–10 min, 80% ethanol; 10–45 min, 80–40% ethanol. The flow rate was 80 mL/min. The detection wavelength was 210 nm. The eluent of 3–4 min was concentrated and freeze-dried to obtain SHMOs.

### Animal NEC model and treatments

Timed pregnant Sprague–Dawley rats were obtained from Laboratory Center of Nantong University and allowed to deliver naturally. Immediately after birth, the newborn rats were separated from the dam and divided into three groups: control (n = 8), NEC group (n = 8) and SHMOs + NEC group (n = 8). All the rats were warmed in an incubator at 37 °C and hand-fed with 0.2 mL special formula milk (3 times per day) using IV catheter (24G) without stylet. The formula consisted of 15 g of PreNAN (Nestle, Germany) in 75 mL of Esbilac milk replacer for dogs (Pet-Ag, USA), which was based on a previous study [9]. The rats in SHMOs + NEC group were hand-fed with formula containing pooled SHMOs at 1500 mg/L which was according to the physiological concentration range of SHMOs in human breast milk [27]. The experimental NEC was induced by exposure to 10-min cold stress (4 °C) and 10-min hypoxia (5%O_2_, 95% N_2_) thrice per day for 72 h. The experiment was terminated at 72 h, the surviving rats were sacrificed, and their terminal ileum tissues were collected for histopathologic examination and further biochemical analysis.

### Histopathologic evaluation

The rat ileum samples were fixed with formalin and embedded in paraffin, followed by cut into 3-μm-thick slices for HE staining and microscopic evaluation as mentioned above. All the samples were graded based on the histopathological findings: Grade 0, normal and intact intestinal architecture without lesions; Grade 1, intact villi with sloughing epithelium; Grade 2, destruction on the upper halves of the villi; Grade 3, destruction extended to the lower halves of the villi with crypts intact; Grade 4, completely destruction of epithelial architecture with or without intestinal wall necrosis or perforations [28]. The score was determined according to the highest score in a slice. Samples graded equal to or higher than 2 were identified as NEC positive. The histopathologic evaluation was done by two pathologists blindly.

### ELISA for IL-1β, TNF-α and IL-6

Concentrations of IL-1β, interleukin-6 (IL-6) and tumor necrosis factor-α (TNF-α) in rat intestinal homogenates were measured by ELISA kits (Abcam, UK), according to the manufacturer’s instructions. The total protein content was quantified by BCA method.

### Western blots

Nuclear and cytoplasmic fractions were collected respectively using Nuclear and Cytoplasmic Protein Extraction Kit (Beyotime, China). Membrane proteins were extracted using the Membrane Protein Extraction Kit (Beyotime, China). Sample proteins were subjected to separation by SDS-PAGE, transferred to polyvinylidene difluoride membranes and blocked with 5% non-fat milk in TBST, followed by being blotted with primary antibodies and HRP-conjugated secondary antibodies accordingly. The primary antibodies used were: mouse monoclinic anti-TLR4 antibodies (Santa Cruz, USA), rabbit polyclonal anti-NLRP3 (Abcam, UK) and mouse monoclonal anti-caspase-1 antibodies (Santa Cruz, USA), rabbit polyclonal anti-NF-κB p65 antibodies (Abcam, UK), rabbit polyclonal anti-phospho-NF-κB p65 antibodies (Abcam, UK), mouse monoclonal anti- IκB-α antibodies (Santa Cruz, USA). Enhanced chemiluminescence (ECL) was to develop the signals. The images were captured using a ChemiDoc™ CRS + Molecular Imager (Bio-Rad, USA) and quantified by Image Lab software (Bio-Rad, USA).

### Cell culture and treatment

Human colonic epithelial cells (Caco-2 cells, obtained from the CellBank of Type Culture Collection of Chinese Academy of Sciences) were cultured in Dulbecco's Modified Eagle's Medium (DMEM) (GIBCO, USA) supplemented with 20% fetal bovine serum, 1% penicillin–streptomycin, and 1% Glutamax (GIBCO, USA) and 1% non-essential amino acids at 37 °C in 5% CO_2_. Caco-2 cells were seeded (2.5 × 10^5^/mL) in 6- or 96-well plates and treatments were conducted when 75% confluence. Various concentrations of LPS (0, 1, 2, 5, 10, 20 μg/mL) or/and pooled SHMOs (500, 1500, 4500 mg/L) were added in serum free DMEM according to experimental design.

### Cell viability assay (MTT method)

Caco-2 cells were seeded (2.5 × 10^4^ cells/well) in a 96-well plate and cultured with or without SHMOs (500, 1500, 4500 mg/L) for 1 h, and then co-cultured with LPS (0, 1, 2, 5, 10, 20 μg/mL) for another 20 h. The supernatants were collected for subsequent gelatin zymography assay; the cells were incubated with 5 mg/mL MTT for another 4 h, followed by addition of 100 μL DMSO to dissolve the formazan crystals. The optical densities (OD) were read by micro-plate reader (Bio-Rad, USA) at 570 nm. The relative grow rates (RGR%) were calculated as cell viability.

### Gelatin zymography analysis for matrix metalloprotease 2 (MMP-2) activity

20μL of cell supernatants were prepared with non-reducing SDS sample buffer (1:1) and subjected to 10% gel containing 0.1% gelatin for electrophoresis. The gels were then washed with 2.5% Triton X-100 for 30 min and incubated in developing buffer (50 mM Tris-base, 0.2 M NaCl and 5 mM CaCl_2_) overnight at 37 °C with gentle shaking. The gels were then washed and stained with Coomassie Brilliant Blue R-250; the images were recorded by camera Gel Doc XR + (Bio-Rad, USA). The intensities of the bands in gels were determined and semi-quantified using the Image Lab software (Bio-Rad, USA).

### Flow cytometry for cell cycle analysis

Caco-2 cells (1 × 10^5^ cells/well) were seeded in 24-well plates and pre-cultured with or without SHMOs (1500 mg/L) for 1 h, followed by treatment with LPS (20 μg/mL) or PBS for 20 h and then fixed in 70% ethanol at 4℃ overnight. Cells were resuspended in PBS containing propidium iodide (PI, 50 μg/mL, Sigma) at 4℃ for 30 min. Cellular DNA content for analysis of cell cycle distribution, was measured using flow cytometry (BD Accuri™ C6-plus, USA). The separation of cell populations in different cell cycle phases was performed using ModFit LT 5.0 software (Verity Software House, USA).

### Mitochondrial membrane potential measurement

Mitochondrial membrane potential determination was performed using mitochondrial membrane potential assay kit (Beyotime, China) according to manufacturer’s instructions. The fluorescence distributions of JC-1 monomeric forms (Ex490nm/Em530nm) and aggregates (Ex525nm/Em590nm) were measured using a fluorescent spectrophotometer (Perkin Elmer, USA).

### Statistical analysis

Data are presented as mean ± SD. Multiple group comparison of continuous data was performed using one-way ANOVA followed by Bonferroni’s or Turkey’s multiple comparisons test. Morphological changes and IHC results between groups were compared using Kruskal–Wallis test. The statistical analysis was conducted using GraphPad Prism 8 (GraphPad Software Inc., USA). A statistical significance was presumed as *p* < 0.05.

## Results

### Increased density of NLRP3 and caspase-1 positive cells were found in the damaged intestinal biopsies from NEC infants

**Fig. 1:**
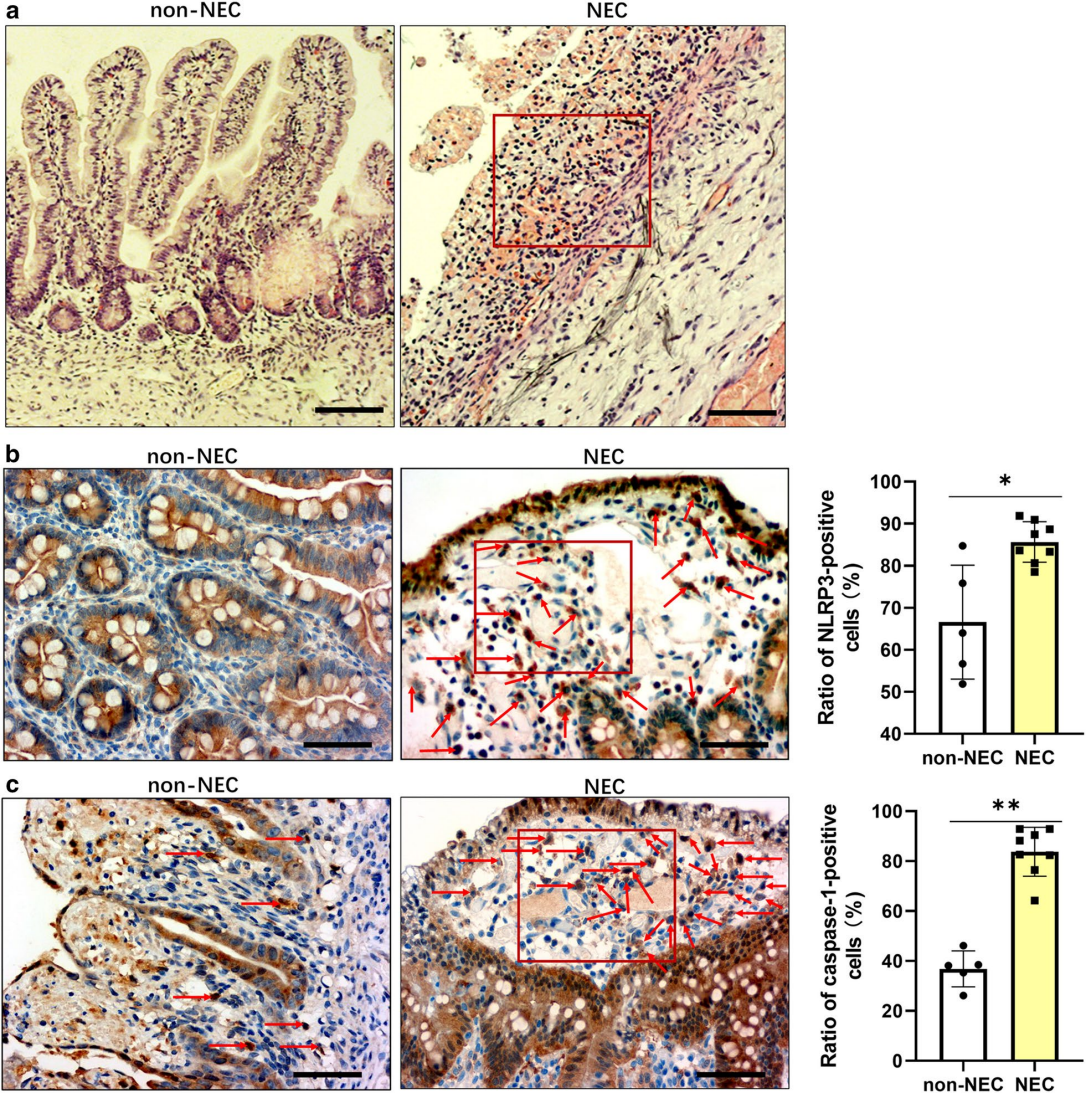
Morphology and expression profile of NLRP3 and caspase-1 in damaged ileum segments from NEC infants. Representative **a** HE staining (100×), immunohistochemistry staining (400×) and quantification of **b** NLRP3 and **c** caspase-1-positive cells in ileum biopsies from NEC and non-NEC infants (aged from 1 to 28 days). Red boxes in the immunohistochemistry images indicates necrotic areas in NEC; red arrows indicate NLRP3-positive and caspasa-1-positive cells in the lamina propria. Scale bars represent 200 µm for 100× or 50 µm for 400 × magnification. Each scatter in the quantification diagram represents the mean value of proportions of positively stained cells from 5 random reading areas in each section at 400× magnification. The lines indicate the mean with SD (non-NEC group, n = 5; NEC group, n = 8). *p < 0.05, **p < 0.01, Kruskal–Wallis test

The HE staining results showed that there were significant histopathological changes, loss of mucosal epithelium, extensive hemorrhagic inflammatory necrosis and massive leucocytes infiltrations in the damaged ileum segments from NEC infants compared to that in non-NEC infants (Fig. 1a). NLRP3 and caspase-1 positive cells were mainly in the epithelial layers and in the lamina propria, which were seen in both NEC and non-NEC samples (Fig. 1b, c). The proportions of NLRP3 and caspase-1 positive cells in the damaged ileum segments from NEC infants were higher than that from non-NEC infants.

### SHMOs supplementation attenuated pathological damage and suppressed hyper-active TLR4/NF-κB signaling pro-inflammatory pathway in NEC rats

**Fig. 2:**
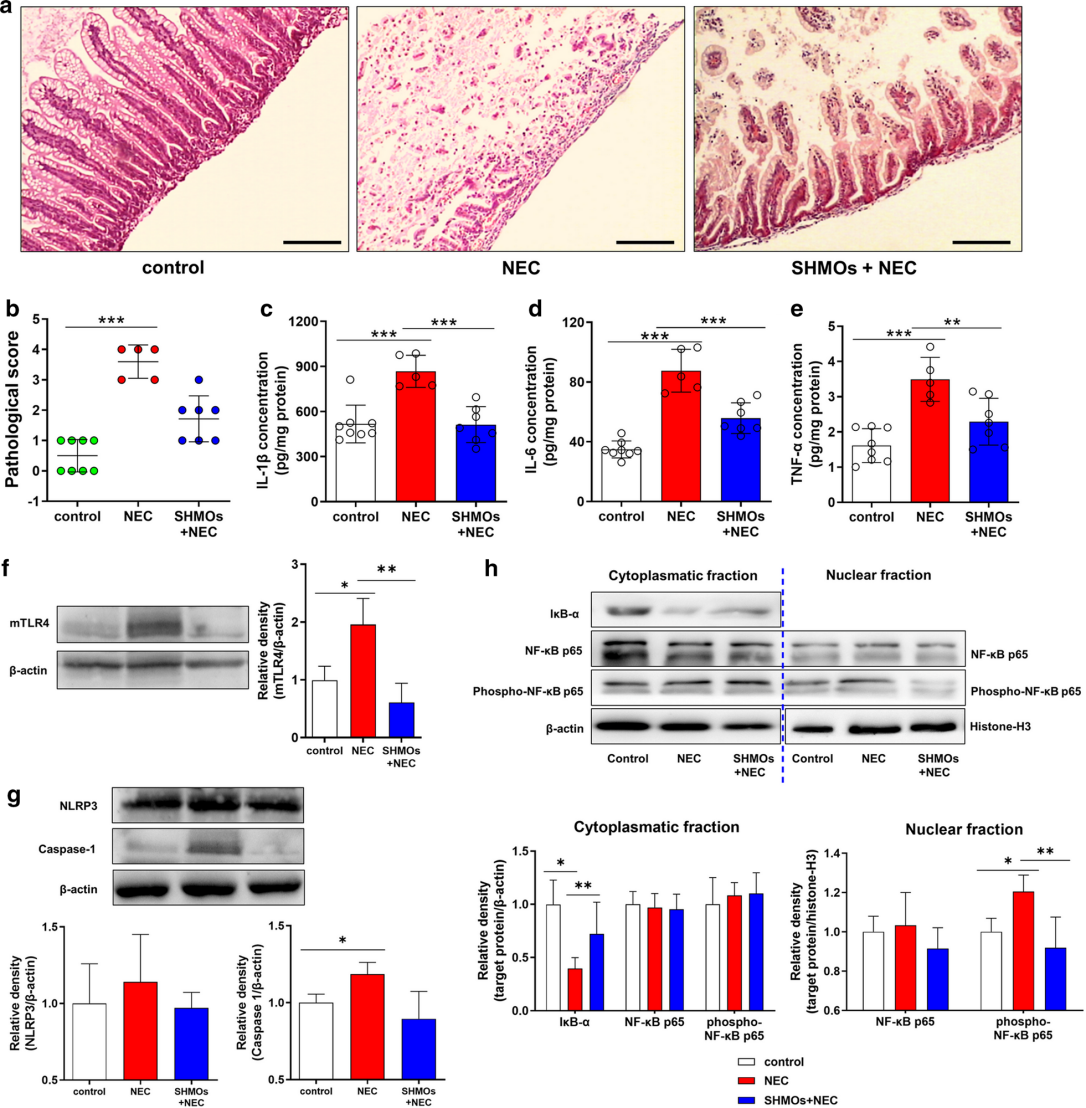
SHMOs prevented pathological damage and TLR4 mediated inflammatory cytokines release in NEC rats. **a** Histomorphology (HE staining, 100×) and **b** pathological scores of rats. Three groups of rats: (1) control group, rats fed with formula; (2) NEC group, rats fed with formula without SHMOs and exposed to 10-min hypoxia (5%O_2_, 95% N_2_) and 10-min cold stress (4 °C) thrice per day; (3) SHMOs + NEC group, rats fed with formula containing SHMOs (1500 mg/L) and exposed to hypoxia/cold stress. After 72 h, ileum samples of all surviving rats (control group, n = 8; NEC group, n = 5; SHMOs + NEC group, n = 7) were collected and examined. Scale bar represents 200 µm. Ileum **c** IL-1β, **d** IL-6 and **e** TNF-α concentrations were determined. The expression of **f** membrane TLR4 (mTLR4), **g** NLRP3 and caspase-1, as well as **h** IκB-α, NF-κB p65, phosphor-NF-κB p65 and nuclear NF-κB p65, phosphor-NF-κB p65 in the ileum of rats. Data were presented as mean ± SD. *p < 0.05; **p < 0.01; ***p < 0.001 analyzed by Kruskal–Wallis test (**b**) or one-way ANOVA followed by Bonferroni’s multiple comparisons test (**c**–**h**)

At the endpoint of experiment (72 h), survival rates (alive/all) of groups were 100% (8/8) in the control group, 62.5% (5/8) in NEC group and 87.5% (7/8) in SHMOs + NEC group. Histomorphological examination of terminal ileum from rats showed that in NEC rats, the intestinal mucosal structure especially epithelium layer was disrupted, muscularis layer was thinner, and leucocytes were infiltrated in to the local sites, which was distinct from that observed in control group (Fig. 2a). The integrity of epithelial layer and inflammatory infiltration in mucosae were attenuated in SHMOs + NEC group compared to that in NEC group. The NEC pathological score of NEC group (3.60 ± 0.55) was significantly higher than that in control group (0.50 ± 0.53) and SHMOs + NEC group (1.71 ± 0.76) (Fig. 2b). Ileum concentrations of IL-1β, IL-6 and TNF-α in NEC group were 1.7 fold, 2.5 fold and 2.2 fold, respectively higher than that in control group (Fig. 2c–e). SHMOs supplementation led to a significant decrease in concentrations of IL-1β, IL-6 and TNF-α in NEC rats.

In NEC group, the ileum expression of membrane TLR4 (mTLR4), NLRP3 and caspase-1 were higher than that in control group (Fig. 2f–g). That was accompanied by a decline in the cytoplasmic IκB-α content and an elevation of NF-κB p65 subunit phosphorylation in the nuclear fraction but not in the cytoplasmic fraction of ileum (Fig. 2h). In SHMOs + NEC group, mTLR4, NLRP3 and caspase-1 in the ileum were reduced, compared with that in NEC group. Meanwhile, cytoplasmic IκB-α was restored, nuclear phospho-NF-κB p65 was decreased and total NF-κB p65 remained stable in both cytoplasmic and nuclear fractions of ileum from rats in SHMOs + NEC group than that in NEC group.

### SHMOs pre-treatment modulated cell viabilities, mitochondrial lesions and MMP-2 hyper-activity in LPS stimulated Caco-2 cells

**Fig. 3:**
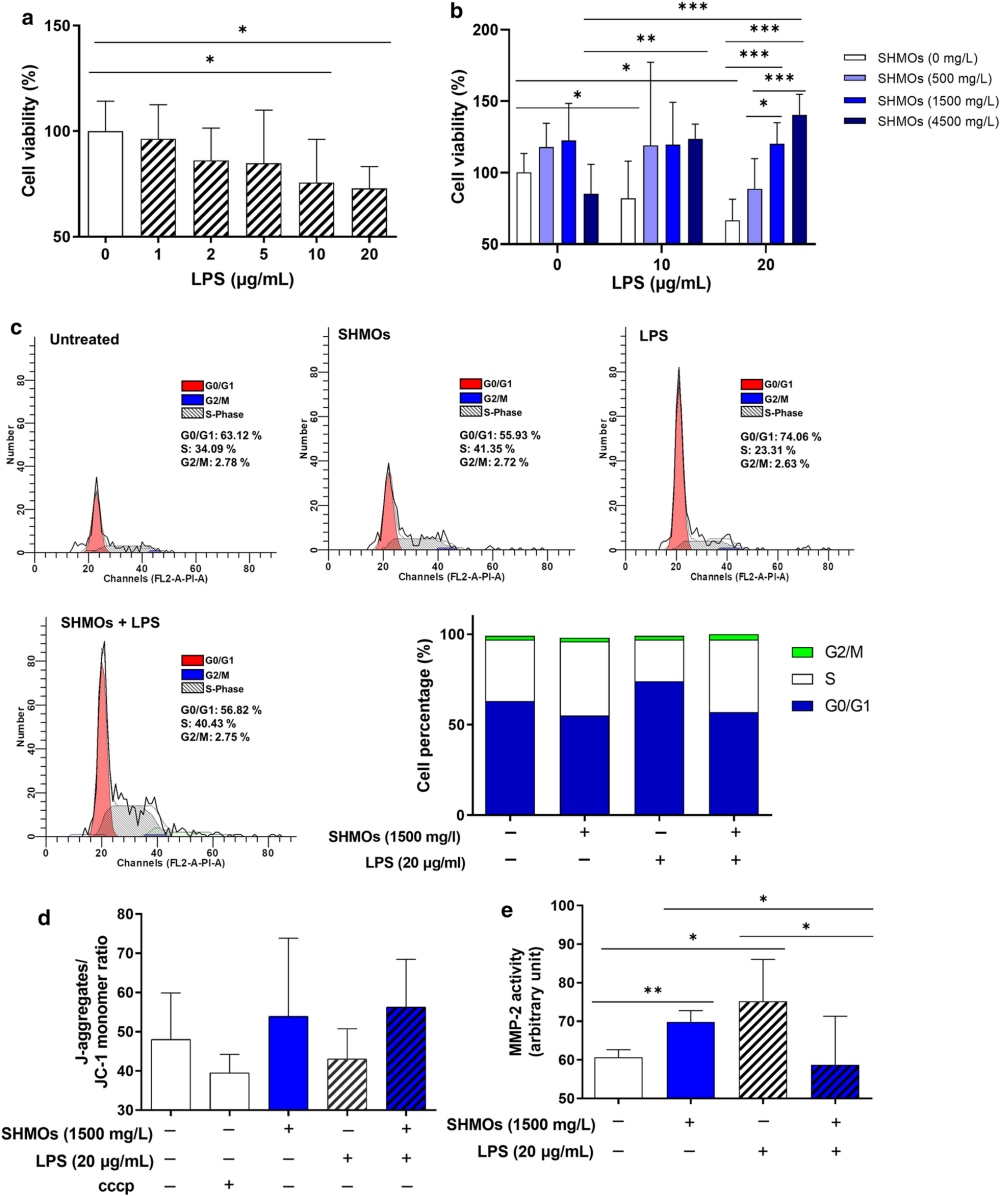
SHMOs pre-treatment promoted proliferation, reduced mitochondrial damage and altered MMP-2 activity of Caco-2 cells. **a** Viabilities of Caco-2 cells co-cultured with different doses of LPS (0, 1, 2, 5, 10, 20 μg/mL) for 20 h. **b** Cell viabilities of Caco-2 cells pre-treated with different doses of SHMOs (500, 1500 or 4500 mg/L) for 1 h, followed by co-cultured with LPS (0, 10 or 20 μg/mL) for another 20 h. **c** Cell cycle, **d** mitochondrial membrane potential and **e** MMP-2 activity of Caco-2 cells pre-treated with pooled SHMOs (1500 mg/L) for 1 h, followed by co-cultured with LPS (20 μg/mL) for another 20 h. Data were presented as mean ± SD (n = 5 for **a** and **b**, n = 3 for **c**–**e**). *p < 0.05; **p < 0.01; ***p < 0.001 analyzed by one-way ANOVA followed by Turkey’s multiple comparisons test

LPS at 1, 2, 5, 10, 20 μg/mL showed dose-dependent inhibition on cell viabilities in Caco-2 cells (Fig. 3a). LPS at 10 and 20 μg/mL significantly reduced cell viability compared with untreated cells, and LPS at 20 μg/mL was used to test the effects of SHMOs. The data from MTT assay showed that Caco-2 cell viabilities were slightly increased by SHMOs of 500 and 1500 mg/L, and pre-treatment of SHMOs at 1500 and 4500 mg/L significantly recovered the cell growth inhibition induced by LPS at 20 μg/mL (Fig. 3b). Cell cycle analysis using flow cytometry showed that compared with untreated Caco-2 cells, LPS-treated cells were arrested at G0/G1 phase, the cell percentage at G0/G1 phase was increased while cell percentage at S phase was decreased by LPS (Fig. 3c). SHMOs pre-treatment mitigated cell cycle arrest by increasing cell percentage at S phase compared with LPS treated cells. Isolated SHMOs caused an elevation in cell percentage at S phase and a reduction at G0/G1 phase.

Mitochondrial membrane potential (ΔΨm) is an indicator of mitochondrial pathway-associated apoptotic cell death. It was observed that the ratio of J-aggregates/JC-1 monomer in LPS-treated Caco-2 cells were reduced than untreated cells (Fig. 3d), indicating loss of mitochondrial membrane potential. This reduction by LPS was attenuated by SHMOs pre-treatment, while SHMOs only also led to an increase in mitochondrial membrane potential compared with parallel untreated cells. Gelatin zymography results showed that the activity of MMP-2 in the supernatant of Caco2 cells treated with LPS was elevated to 1.24 fold higher than that in untreated cells (Fig. 3e). SHMOs caused a marked reduction by 26.4% in MMP-2 activity in the presence of LPS. SHMOs led to a significant increase in MMP-2 activity than that in untreated cells.

## Discussion

The incidence of NEC is continuously increasing due to the improved early survival of newborns while the mortality rate of NEC does not change due to limited preventive and therapeutic approaches [29]. Although human milk is long known to be effective measures to prevent NEC, most of mothers giving birth to premature infants can’t produce sufficient milk [30]. It is of great importance to explore the effective compounds in human milk and their mechanisms to trace the pharmaceutical targets against NEC. In this study, our results suggested that SHMOs-supplemented neonatal rats were less susceptible to develop NEC and this protection was associated with inhibitory regulation of SHMOs on TLR4/NF-κB/NLRP3 signaling pathway.

Our morphology and immunohistochemistry examination on human infants’ ileum tissue sections illustrated that NLRP3 and caspase-1 were primarily expressed in the cytoplasmic space of mucosal enterocytes and cells in the lamina propria beneath the epithelium. As reported, intestinal NLRP3 is expressed mostly in both immune and epithelial cells [31], which is consistent with our immunohistochemistry observation. The frequencies of NLRP3 positive and caspase-1 positive cells were higher in the inflamed intestinal segments from NEC infants than that from age-matched non-NEC infants. This indicated the involvement of NLRP3 inflammasome in NEC pathology. Also, our finding is in accordance with and supports current opinions that NEC is developed as consequence of an inappropriate hyper-responsiveness to perinatal insults including bacterial colonization in premature gut [15].

Although SHMOs showed anti-NEC potential in previous preclinical studies and the beneficial effects of SHMOs have been explored in infantile undernutrition and in specific bacterial growth [13, 32], the mechanisms of SHMOs in preventing NEC are largely unknown. In our neonatal rat model of NEC induced by formula-feeding/hypoxia/cold stress, clinical NEC-like lesions including intestinal epithelium disruption, inflammatory leucocytes infiltrations, thinner muscularis layers, necrosis and perforations, were observed. SHMOs at the concentration of 1500 mg/L, which is within the physiological range as reported in human milk, improved rat survival and prevented the intestinal inflammatory lesions in response to hypoxia/cold stress as indicated by decreased NEC scores and ileum concentrations of IL-1β, IL-6 and TNF-α. mTLR4 transmits the pro-inflammatory signal into the cytoplasm and leads ultimately to the activation of NF-κB and subsequent production of cytokines [33]. Western blotting analysis showed that SHMOs pre-treatment suppressed expression of mTLR4 and downstream translocation of phosphorylated NF-κB p65, the active form of NF-κB, in the ileum of NEC rats. IκB-α, whose rapid proteolysis in the cytoplasm is necessary for NF-κB activation in the inflammatory cascade initiation, was reduced in the ileum of NEC rats and restored in response to SHMOs. The increase of ileum protein expressions of NLRP3 and caspase-1 were seen in NEC rats without SHMOs supplementation, but not with rats supplemented with SHMOs. The results from this study suggested that SHMOs prevents neonatal rats from NEC-related damages by targeting TLR4/NF-κB/NLRP3 inflammatory pathway *in-vivo*.

Epithelial barrier disruption and abnormal epithelial cell death initiate the early stage of NEC and are required for further development of overt NEC [34]. Epithelial cell proliferation plays an important role in epithelial cell replenishment and repair during wound healing, functioning for maintaining epithelial integrity and homeostasis [35]. The protection by SHMOs in-vivo was supported by our data from LPS-induced inflammatory model in human epithelial Caco-2 cells. Results from this study showed that TLR4 activator, LPS, dose dependently suppressed Caco-2 cell viability and this suppression was associated with LPS-caused cell arrest at G0/G1 phase. SHMOs (1500 mg/L) pre-treatment increased cell percentage at S phase and promoted cell growth in LPS-stimulated Caco-2 cells. This present study showed that SHMOs promoted Caco-2 cell proliferation during external challenge, indicating SHMOs can prevent epithelial barrier damage in the initial stage of NEC development. It was reported that mitochondrial dysfunction was associated with NLRP3 activation and IL-1β production [36] and was responsible for intestinal epithelial cell death during NEC development [37]. Resting NLRP3 localizes in endoplasmic reticulum prior to stimulations, whereas on inflammasome activation, NLRP3 and its adaptor redistribute to the perinuclear space around mitochondria organelle clusters and endoplasmic reticulum [38]. NLRP3 inflammasome senses mitochondrial dysfunction and responses by activation [38]. In our Caco-2 inflammation model, it was observed that the mitochondrial membrane potential of Caco-2 cell was reduced in response to LPS and was restored by SHMOs during LPS stimulation. Moreover, LPS and SHMOs were found to show regulatory effects on MMP-2 activities. MMP-2 is an important matrix metalloproteinase responsible for degradation of extracellular matrix proteins during wound healing and cell migration. Increased MMP-2 expression occurs after injury and can serve as an indicator of prolonged wounds [39]. Our results from gelatin zymography analysis showed that LPS only induced higher activity of MMP-2 in the cell culture supernatant than that in untreated cells. SHMOs pre-treatment significantly decreased activity of MMP-2 in LPS-stimulated Caco-2 cells. These results indicated that pooled SHMOs mixture could attenuate LPS-induced alteration on enterocytes migration and promote epithelium regeneration and repair, and thus improving the resistance of epithelial barrier against external and internal challenges in early life.

## Conclusions

**Fig. 4:**
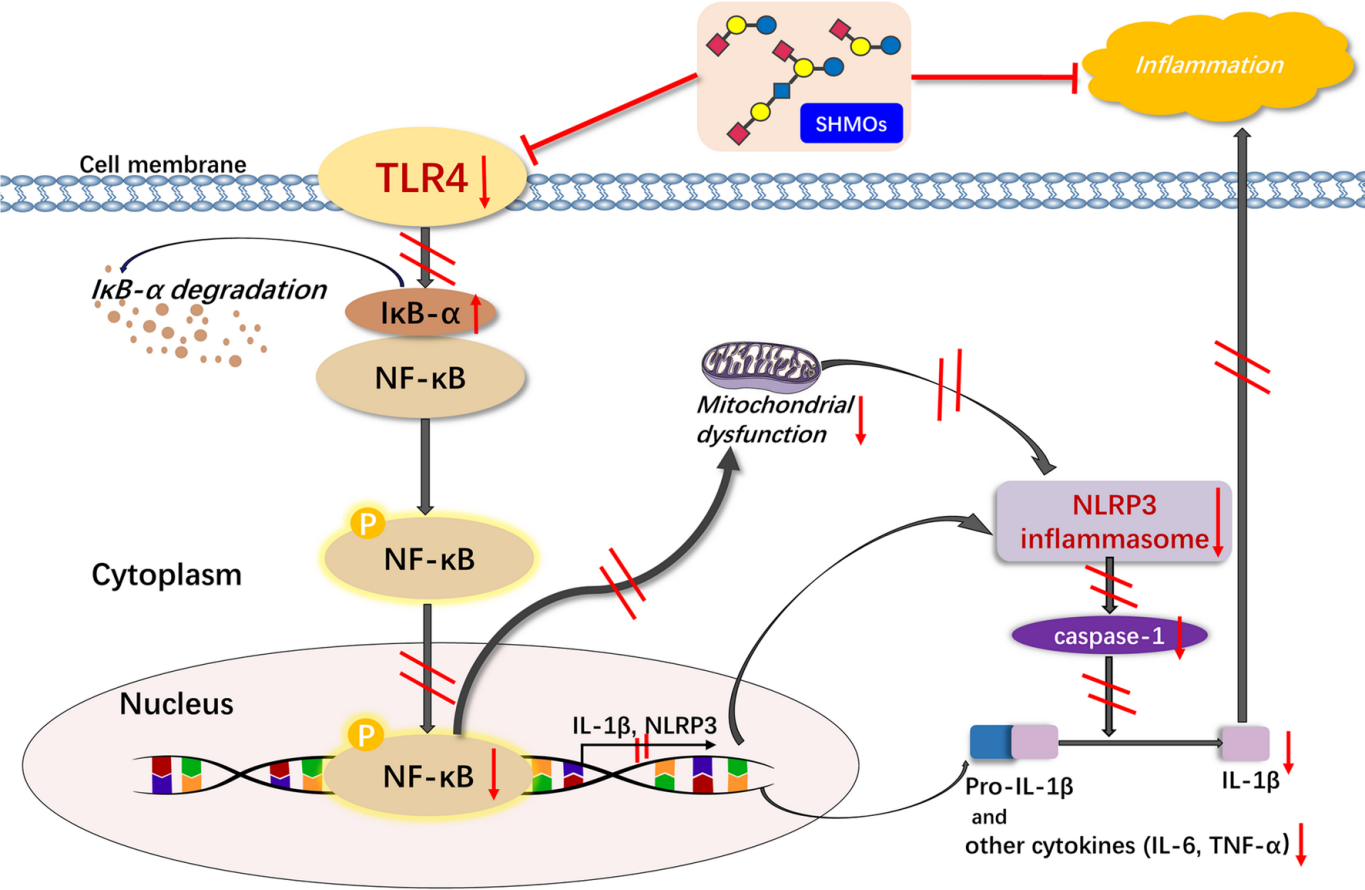
Schematic diagram of SHMOs exerting anti-inflammatory effects in NEC. Pooled SHMOs mixture attenuated hyper-expressed TLR4, inhibited NF-κB-mediated production of inflammatory cytokines, attenuated mitochondrial dysfunction and NLRP3 activation, and thereby mitigated inflammatory injury in NEC. The red arrows indicate the change of signaling molecules after exposure to SHMOs; the red lines indicate the potential steps that SHMOs might affect in the pathway. IκB-α, inhibitor κB-α; IL-1β, interleukin-1β; IL-6, interleukin-6; LPS, lipopolysaccharide; NF-κB, nuclear factor-κB; NLRP3, nod-like receptor pyrin 3; TNF-α, tumor necrosis factor-α; TLR4, toll like receptor 4

In conclusion, this study (1) provided evidence of increased frequencies of NLRP3 and caspase-1 positive cells in the lamina propria of damaged intestinal areas in NEC infants; (2) showed SHMOs inhibited TLR4/NF-κB/NLRP3 signaling pathway, suppressed inflammatory cytokines (IL-1β, TNF-α and IL-6) production and reduced NEC incidence and pathological damages in inflamed ileum of NEC rats in-vivo; (3) suggested SHMOs promoted epithelial cell proliferation, restored mitochondrial membrane potential and regulated MMP-2 activities in LPS-stimulated Caco-2 cells *in-vitro*. This study provides clinical evidence of involvement of NLRP3 inflammasome in pathology of NEC and indicates that SHMOs prevents over-activation of TLR4/NF-κB/NLRP3 signaling pathway (Fig. 4), thereby protecting newborn rats and epithelial cells from inflammatory damages.

## Data Availability

All data relevant to the study are included in the article, or available upon contacting the corresponding author.

## Abbreviations

GOS: Galacto-oligosaccharides
HE: Haematoxylin and eosin
HMOs: Human milk oligosaccharides
IκB-α: Inhibitor κB-α
IL-1β: Interleukin-1β
IL-18: Interleukin-18
IL-6: Interleukin-6
LPS: Lipopolysaccharide
MMP-2: Matrix metalloproteinase-2
mTLR4: Membrane toll like receptor 4
NEC: Necrotizing enterocolitis
NF-κB: Nuclear factor-κB
NLRP3: Nod-like receptor pyrin domain-containing 3
SHMOs: Sialylated human milk oligosaccharides
TNF-α: Tumor necrosis factor-α
TLR4: Toll like receptor 4
